# Mobile Access to Medical Records in Heart Transplantation Aftercare: Mixed-Methods Study Assessing Usability, Feasibility and Effects of a Mobile Application

**DOI:** 10.3390/life12081204

**Published:** 2022-08-08

**Authors:** Julia Müller, Lina Weinert, Laura Svensson, Rasmus Rivinius, Michael M. Kreusser, Oliver Heinze

**Affiliations:** 1 Institute of Medical Informatics, Heidelberg University Hospital, Im Neuenheimer Feld 130.3, 69120 Heidelberg, Germany; 2 Section for Translational Health Economics, Department for Conservative Dentistry, Heidelberg University Hospital, Im Neuenheimer Feld 400, 69120 Heidelberg, Germany; 3 Department of Cardiology, Heidelberg University Hospital, Im Neuenheimer Feld 410, 69120 Heidelberg, Germany; 4 German Center for Cardiovascular Research (DZHK), Partner Site Heidelberg/Mannheim, 69120 Heidelberg, Germany

**Keywords:** electronic health records and systems, feasibility, mHealth, mixed-methods, mobile application, patient self-management, usability

## Abstract

Background: Patient access to medical records can improve quality of care. The phellow application (app) was developed to provide patients access to selected content of their medical record. It was tested at a heart transplantation (HTx) outpatient clinic. The aims of this study were (1) to assess usability of phellow, (2) to determine feasibility of implementation in routine care, and (3) to study the effects app use had on patients’ self-management. Methods: Usability was measured quantitatively through the System Usability Scale (SUS). Furthermore, usability, feasibility, and effects on self-management were qualitatively assessed through interviews with users, non-users, and health care providers. Results: The SUS rating (*n* = 31) was 79.9, indicating good usability. Twenty-three interviews were conducted. Although appreciation and willingness-to-use were high, usability problems such as incompleteness of record, technical issues, and complex registration procedures were reported. Improved technical support infrastructure, clearly defined responsibilities, and app-specific trainings were suggested for further implementation. Patients described positive effects on their self-management. Conclusions: To be feasible for implementation in routine care, usability problems should be addressed. Feedback on the effect of app use was encouraging. Accompanying research is crucial to monitor usability improvements and to further assess effects of app use on patients.

## 1. Introduction

Transparency in health care can improve patients’ understanding and engagement [1,2,3]. Giving patients access to their medical records can (1) enhance health literacy, (2) improve adherence to therapy, (3) increase patients’ health-related self-care, (4) improve doctor–patient communication, and (5) enhance quality of care [4,5,6,7,8,9,10,11]. Patient portals can be used to provide patients with their medical records [12].

Encouraging patients to become more actively engaged in their health care holds several benefits. This is especially true for chronically ill patients requiring ongoing self-management and frequent check-ups [13], for example after orthopedic hip surgery [14] or after heart transplantation (HTx). After HTx, patients need long-term aftercare at highly specialized HTx centers to enable fullest possible reintegration into normal life as well as early detection of deterioration in health status or risks [15,16]. Patients therefore usually follow a specific immunosuppressive protocol to avoid graft rejection. The immunosuppressive drug therapy must be guided by therapeutic drug monitoring in order to prevent over- or underdosing which may lead to adverse effects (hand tremor, loss of hair, and kidney failure). In addition, side-effects such as arterial hypertension, hyperlipidemia, diabetes, malignancies, and osteoporosis may occur and need to be treated adequately [15,16]. Hence, drug trough levels have to be closely monitored via periodical blood withdrawals as they are often not within target range. If drug trough levels are not within target range, the immunosuppressive drug therapy has to be adapted accordingly and patients need to be informed about changes in medication as soon as possible; however, notification of patients using conventional means of communication such as mail or telephone takes time and can cause a serious delay. Consequences arising out of delay can range from severe kidney failure due to overdosing to irrecoverable graft failure as a result of underdosing [17].

Use of modern communication technologies, facilitation of patient participation, and granting patients access to their own medical records using a safe, secure online platform could result in improved post-transplant survival and quality of life in patients after HTx. It could also increase patient satisfaction with their treatment, since they can participate in their care process more actively and could gain flexibility due to not having to wait for calls or letters to see their drug trough levels.

### 1.1. Study Context

Although entitled by national law in Germany, only a few patients access information stored in electronic health records (EHR) [18,19]. At the Heidelberg University Hospital, Heidelberg, Germany, the prerequisite for a patient-accessible medical record was met by implementing a web-based personal, cross-institutional health record, named “PEPA”, in 2016 [20]. PEPA provides the technical infrastructure for enabling patients to manage their providers’ access to the patient health record [20,21]. Patients gain access to their PEPA through a mobile application (app) called “phellow” which was developed within the start-up project “phellow seven”. Phellow can be coupled with PEPA and content from the patient portal can be viewed on a private mobile device [22]. The extent to which patients can access data from the PEPA can be customized by the treating organization/clinic. The implementation of phellow into clinical routine was tested in a first use case with patients after HTx at the Department of Cardiology, Angiology and Pneumology, Heidelberg University Hospital, Germany from late 2018 onward [22,23]. For this cohort, making drug trough levels accessible via app was considered especially promising. Patients using the app can adjust their immunosuppressive medication immediately when triggered by most recent laboratory results. Prior to using phellow, an onboarding process must be performed in which patients receive a letter with an initial password to couple PEPA with phellow. Then, the app has to be downloaded and installed on a smartphone. Afterwards, PEPA and phellow can be coupled following a secure authentication process [22].

To assess the feasibility of further implementation of the app into routine care in other departments at our institution and to evaluate usability of the application, a structured mixed-methods evaluation was conducted.

### 1.2. Objectives

To assess the feasibility of further implementation of the app into routine care in other departments at our institution and to evaluate usability of the application; a structured mixed-methods evaluation was necessary. The aims of our study were (1) to assess usability of phellow from the perspective of patients who tested the app; (2) to determine if phellow is feasible to be further implemented in routine care from the perspective of both patients and health care providers (HCP) and (3) to study the self-reported effects the use of phellow had on patients’ self-management.

## 2. Materials and Methods

### 2.1. Study Design

This study was approved by the Ethics Committee of Heidelberg University Hospital (S-492/2020). A mixed-methods design was chosen to combine the strengths of qualitative studies (e.g., the ability to cover a broad thematic spectrum and account for individual experiences) and quantitative studies (e.g., the ability to compare usability test scores with benchmarks). Participants were:

- Non-physician (e.g., medical assistants) and physician HCPs at the HTx outpatient clinic of the Heidelberg University Hospital,
- Patients after HTx who actively and independently used phellow (in the following referred to as “active users”),
- Patients after HTx who had used phellow independently but quit app use (in the following referred to as “non-users”).

**Figure 1:**
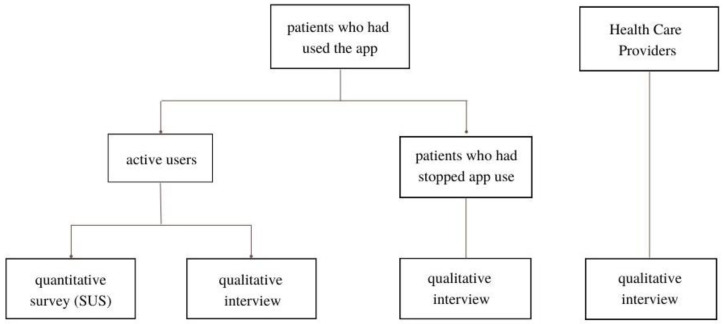
Study flow chart.

General criteria for inclusion in the study were age (>18 years), fluency in either German or English, and ability to give informed consent. Confidentiality and anonymity were ensured throughout the entire study. The study was reported according to the “Consolidated criteria for reporting qualitative research” checklist (Supplementary Materials Additional File S1) [24]. A study flow chart is presented in Figure 1.

#### 2.1.1. Quantitative Measures

To assess usability, the System Usability Scale (SUS) [25] in a non-validated, translated version, was used [26]. It was chosen because of its extensive use in medical research, simplicity, and suitability for small sample sizes [26,27,28]. Even though the SUS was originally designed as a one-dimensional instrument to measure usability, eight of the questions address a usability dimension while two reflect a learnability factor [29]. In our study, the SUS was complemented by a set of questions on app and internet use habits and sociodemography developed by the authors. Study data were collected and managed using REDCap (Research Electronic Data Capture) tools [30,31] hosted at the Heidelberg University Hospital.

Only active users participated in the quantitative part of the study. This decision was made to minimize recall bias (non-users) and due to very little interaction with the app’s user interface (HCPs).

#### 2.1.2. Qualitative Measures

Additionally, we conducted open-ended, semi-structured, guide-based interviews. Specific guides were developed for every participant subgroup. The interview guides (Supplementary Materials Additional file S2) were based on theoretical considerations and an extensive literature search and pre-tested by a group of interprofessional researchers (Health Services Research, Medical Informatics). A sociodemographic questionnaire was used to collect data on interview participant characteristics (Supplementary Materials Additional file S3).

### 2.2. Data Collection and Analysis

#### 2.2.1. Quantitative Measures

In-person recruitment took place from September to November 2020. Recruitment of active phellow users was supported by medical staff at the HTx outpatient clinic. To ensure a structured recruitment process, all staff members received instructions and informational material from the study team. During waiting times for routine appointments, patients were offered participation in the survey by the medical staff or the study team. After assessing if the inclusion criteria were met, patients were given a tablet on which they conducted the anonymous online questionnaire. Since not all potential participants had routine appointments during the in-person recruitment phase, all phellow users registered at the HTX outpatient clinic received an email invitation to fill in the survey in their web browser after in-person recruitment ended.

After both recruitment phases were completed, the survey was closed, and data were checked for plausibility. Descriptive and correlation analyses were conducted with R statistical software (version 4.0.4, R Foundation for Statistical Computing, Vienna, Austria).

#### 2.2.2. Qualitative Measures

We chose a purposive sampling method. Patients were approached during waiting times for routine appointments at the clinic. With HCPs, individual appointments were arranged. Participants did not receive reimbursements. Data were collected until saturation of information was reached, which was assessed based on deviant observations and consistency of findings. Face-to-face interviews were conducted by two female researchers (L.W. and J.M.) with a professional background in Health Services Research and profound experience in qualitative interviewing. Non-participants were not present during the interviews. No relationship with participants was established prior to participation. No repeat interviews were carried out and no field notes were taken. Interviews were audiotaped, pseudonymized, and transcribed verbatim. Data were transcribed, managed, and analyzed with MAXQDA Standard 2020 (Version 20.3.0; Verbi software, Berlin, Germany). Transcripts were not returned to participants. Data analysis was conducted according to thematic analysis [32]. First, transcripts were reviewed independently, and themes were identified a priori from literature and the interview guide and inductively de novo from data. Second, discrepancies were discussed in iterative cycles until consensus on themes and the final coding scheme was reached. After analyzing 23 interviews (16 patients and 7 HCPs), data saturation was agreed on as no further themes and no new data occurred. Themes were organized into main- and subthemes. Each theme was defined by a quote from the transcripts (Supplementary Materials Additional file S4).

## 3. Results

### 3.1. Quantitative Measures

The questionnaire was filled in by 31 active phellow users. The participant age ranged from 27 to 71 years (mean 53.7; SD 11.9). Nine out of 31 patients were female (29%). Seventeen patients (54.8%) had used phellow for more than 12 months. Table 1 provides information on the participant characteristics of the quantitative measures.

The average usability rating measured via SUS was 79.9 (SD 14.1). Scores are out of 100 with a higher score indicating higher usability. In this study, the usability dimension had a score of 79.9 and the learnability dimension had a score of 79.8, indicating that patients perceived both usability and learnability of the phellow app similarly as good. Compared with published results using the SUS, a rating of 79.9 places the app above the 86th percentile [33]. Additionally, when drawing attention to the average SUS score of 68 [33], usability of phellow was considered good in this study. When focusing on individual ratings, it could be observed that 8 patients rated usability higher than 90 whereas 3 patients rated below 60. No statistically significant correlation was found between SUS scores and users’ sociodemography or habits of phellow use.

### 3.2. Qualitative Measures

Twenty-three participants were approached and successfully recruited for the interview study. Table 2 provides information on participant characteristics. Interviews were conducted between September and November 2020 in consultation rooms located at Heidelberg University Hospital. Interview duration varied between 10 and 29 min (mean 16).

Data were organized into 4 main themes and 11 associated subthemes. The presentation of results follows the commonly used “Fit between Individuals, Task and Technology” (FITT) framework [34]. As feasibility refers to the appropriateness of an intervention for further implementation and testing [35], the perspectives stated in the FITT framework provide a holistic view on feasibility aspects. Usability aspects are presented according to the ISO 9241-11 framework, which defines usability as “the extent to which a system, product or service can be used by specified users to achieve specified goals with effectiveness, efficiency and satisfaction in a specified context of use” [36]. These are crucial factors in assessing if technology meets user needs [25,36].

All presented interview quotes indicate the respective participant group and transcript position (TP). They were translated from German into English with due diligence.

#### 3.2.1. Feasibility

##### Task

Codes referring to tasks and interactions with the app and the accompanying infrastructure were assigned to the subcategory *task*. Here, HCPs and patients talked about the *clarity of tasks*, for example, whether HCPs knew how to sign-up patients or whether patients could find necessary information. They found tasks clear and easy to perform. HCPs further discussed *perceived fit with clinic workflow*, describing *phellow* as helpful and timesaving in supporting workflows.

*“[…] You open the patient record and say to yourself: “Oh nice, he uses the app” […]. We’re happy about everyone who’s using it. It’s definitely easier.”*

[Medical staff#8, TP48]

Questions were raised about *document access* as it was unclear to patients and HCPs why not all documents were visible. Concerning prospective full implementation in routine care, HCPs wished to withhold or manually release some documents, such as decisions about organ transplantations or oncological therapy. Both patients and HCPs desired more concrete and robust *technical support infrastructures*, such as hotlines or manuals.

##### Individuals

This subcategory encompasses codes referring to experiences, requirements, or views regarding further implementation of phellow into (routine) care. Overall, patients and HCPs reported high general interest in the implementation of phellow. However, generational differences in smartphone ownership and technical capabilities were seen as critical prerequisites. Both patients and HCPs demanded training. HCPs specifically shared that more in-depth training would be necessary to be able to support patients. A further patient-related requirement was that patients would need to have some degree of medical knowledge to facilitate the interpretation of medical documents. Furthermore, patients and HCPs reported that the definition of responsibilities could be clearer. For instance, it was unclear to some HCPs whose responsibility it was to invite patients to use the app. Technical support was provided by both HCPs and the IT department, which led to confusion.

*“I asked them [medical staff] once, but they said they had no time for it at that moment. Another time they told me to contact IT. I also googled. But from all these pages, I didn’t know who the right person would be to contact.”*

[non-user#1, TP24]

##### Technology

Regarding the subcategory technology, which addresses all technological aspects, one patient and one HCP described concerns about the system’s stability. They wondered whether the system could handle the number of documents and data generated in the hospital if the app was to be implemented into routine care. They did not elaborate further on these thoughts.

Due to the overlapping nature of content, further aspects of technology are presented in the following chapter on usability.

#### 3.2.2. Usability

##### Effectiveness

A repeatedly mentioned aspect obstructing the app’s effectiveness was *completeness and timeliness of content*: While drug trough levels were mostly available, patients criticized that physician’s letters (typically containing medical history, examination results and recommendations for drugs and treatments) either did not appear in *phellow* or were accessible only with a large delay.

*Presentation of content* was considered incomprehensible as only the initial creation date of documents was displayed in the app, even though more recent document versions were uploaded. Most commonly reported *technical issues* were missing content or incomplete history of documents and sudden app crashes. Especially non-users emphasized that these technical problems influenced their decision to not use the app any longer.

*“Sometimes it just crashes. I can’t even get to my log-in credentials. But I don’t know what it is related to. Whether it is really because of the app or…”*

[user#17, TP6]

Besides these issues, patients reported that effective use was ensured by a *simple navigation*, which was considered “*clear*” (non-user#2, TP54) and “*self-explanatory*” (user#16, TP28). The app’s *aesthetic design* was perceived as “*professional*” (user#5, TP34) and “*pleasant*” (user#16, TP26).

##### Efficiency

Both users and non-users reported that the app was *easy to understand* without additional help and with minor learning effort. Furthermore, patients described the app as *easy to use.* They expressed that using the app was generally intuitive and easy.

Contradictory positions were shared concerning *registration effort*: Some patients reported that registration and initial log-in were highly complex while others found it simple. Still, both patients and HCPs appreciated that they had saved time since using the app, making overall communication more *efficient*.

*“I think the advantages are that patients don’t have to call us. They get the message directly; we don’t lose any time. That makes a lot of sense. Especially for immunosuppression levels.”*

[physician#9, TP26]

##### Satisfaction

The subcategory satisfaction comprised expressions of personal beliefs about contentment, concerns, and intention of app use. Generally, patients showed a high *appreciation of the system*. Medical staff also valued the system and praised its time-saving effects, for instance, that the number of calls had decreased. HCPs moreover shared *concerns* about overburdening patients with complex medical information. They also expressed a lack of control, because there was no confirmation whether patients have accessed the documents provided via the app. Among patients, few concerns such as loss of data due to technical errors were reported. While the non-users’ *willingness to use* the app was limited because they had encountered problems with registration and log-in, users and HCPs expressed high willingness to use the app for its several benefits.

#### 3.2.3. Effects on Self-Monitoring

Users appreciated newly gained *independence* since they could access recommended changes of drug trough levels on the go, were not dependent on office hours, and did not have to stay home to wait for calls from the hospital. Patients felt *reassured* as they were able to access their medical data anytime.

*“Sometimes, I forget it but, in the evening, before I take my medication, I used to think “Oh, maybe I should have called”. Now, I can just look it up. Or, when I prepare my medication, [I think] “was it like this or like that?”. And, of course, I can then just quickly check my app and see. That’s great.”*

[user#14, TP38]

They furthermore liked that they could *share results* by printing or showing documents in the app to other physicians.

A negative aspect few users noted was the potential loss of personal contact and the inability to ask short questions.

*“…I can’t write something within the app. If it was an email, I would be able to ask a question and receive an answer. But here (in the app), I only get the results and can’t get in touch through the app. Like with emails, where I can write something and ask questions.”*

[user#17, TP52]

#### 3.2.4. Recommendations

##### Recommendations on Improving App Usability

Referring to the *user interface*, many users asked for a chronologically plausible presentation and push notifications. Users furthermore stated that they would like to customize the interface, filter content by date/type of document, and use the app via a web browser. Another repeatedly mentioned aspect by all participants was a simplification of registration and log-in to ensure *ease of use*.

##### Recommendations on New Functionalities

Referring to *interaction and information* aspects, patients requested full access to their complete medical record via phellow. Patients furthermore reported that a communication module within the app could facilitate contact. This was rated positively by the HCPs; physicians, however, anticipated a higher workload. Both groups suggested additional information on medical terms, disease-specific aspects, answers to common technical difficulties, and the possibility of transferring patient records. A recommendation mainly shared by HCPs was the availability of a read confirmation for medical reports as a reassurance that documents were seen by patients. Regarding *organization of therapy,* both groups suggested additional features such as making appointments, requesting medication refills, accessing medication plans, or reminders for medication intake or appointments. Patients and HCPs furthermore wanted to give family or caretakers access to the app. Last, participants suggested to upload *patient-reported data*, such as vital signs, via phellow. Table 3 gives an overview on recommendations for mobile patient portals.

## 4. Discussion

This study provided insights about the implementation of phellow, a mobile application enabling access to specified contents of patient records, at an outpatient clinic of an academic hospital in Germany. Quantitative measures consisted of the SUS questionnaire wherein patients rated usability as good (79.9). The relatively small sample size (*n* = 31) might explain why no statistically significant correlations between SUS scores and patients’ sociodemography or habits of app use were found.

Qualitative data enabled us to discover specified usability aspects as well as valuable recommendations. Accompanying interviews showed that appreciation, interest, and willingness to use were high. However, problems involving technical and organizational barriers interfered with a more widespread implementation of phellow.

Users and HCPs showed gratitude and appreciation for the implementation of phellow in HTx aftercare. Our participants were enthusiastic about further development of the patient portal and gladly suggested additional functions and recommendations. Table 3 provides an overview of these recommendations. We believe that these should be taken into consideration and are highly useful for other medical institutions or developers aiming to develop or implement a (mobile) patient portal.

### 4.1. Comparison with Prior Research

While ease of understanding did not cause problems, ease of use was considered low when technical issues occurred. Former research reports that technical problems can have a major negative influence on ease of use [37,38]. Concerning technical issues, some participants reported that log-in was easy and the initial registration process was manageable, although others found it highly complex. This discrepancy could be explained by participants’ individual expectations and capabilities to manage a multi-step registration process. The multi-step registration process was required for data protection reasons and potentially introduced complexities for participants with less individual experience. Some participants even reported quitting app use because the log-in process was perceived as too cumbersome. Similar problems were identified in studies on patient portals before [12,39]. These findings from literature and our study imply that registration and log-in are of heightened relevance for the decision to use or continue using mHealth applications. Registration and log-in represent the first interaction with an app in both first-time use and regular use, making issues in these steps highly noticeable and potentially irritating. To avoid these issues, some participants requested close guidance or simplified registration/log-in.

As a further barrier to effective use, the record in phellow was considered incomplete and the presentation of content was described as incomprehensible. A reason for this could be the fact that drug trough levels were transferred to the app automatically while physician’s letters were released manually. HCPs emphasized that this preselection of documents was important to avoid the risk of overburdening patients, which is a common concern [4,18,40,41,42]. However, whether the inconsistent availability of physician’s letters in app stemmed from technical issues or selective release remained unclear in this study and requires further investigation. Even though patients reported phellow’s usability was obstructed by the aforementioned issues, their satisfaction with the app and the perceived positive influence on their care was high. Patients in our study especially liked that they had gained independence and that phellow helped them making communication and care processes more efficient. Time-saving effects were likewise reported by HCPs. These effects were reported for patient portals before [12,38,42].

Addressing feasibility requirements, participants demanded clearly defined responsibilities, a comprehensive training for users and HCPs, and a support strategy. Although these are known resource factors important to the implementation of technologies [43,44,45], there is currently no training provided to patients or HCPs. This could have affected both usability and feasibility experiences. Users’ lack of knowledge or poor training might lead to errors which, in turn, can cause frustration [37,38].

The importance of addressing patient-related requirements, such as age or generational differences or the need for basic medical knowledge, is a well-known implementation prerequisite when giving patients access to their medical records [6,40,46,47]. Even though few participants voiced their concerns on these aspects in the current study, most patients reported that they did not face problems in understanding their medical documents. They however stated that explanations of medical terms in the app would be beneficial, which corresponds to former research on patient portals [12,39]. Concerning further implementation of phellow, most patients demanded access to their full record and emphasized that it should be chronologically plausible, enable document upload, and allow shared access with caretakers. Patients’ desire for access to the full medical record was likewise reported in former research on open patient records [4,39,46,47,48,49].

### 4.2. Strengths and Limitations

To our knowledge, this was the first structured usability and feasibility evaluation of an app enabling mobile patient access to medical records in Germany. By including HCPs’, users’, and non-users’ perspectives we gained broad and holistic insights on feasibility and usability. Integrating non-user perspectives facilitated the identification of crucial usability aspects that influenced decisions to quit app use. The evaluation was conducted among users who have used phellow under real-life conditions rather than in a laboratory setting. Hence, participants did not only share perspectives on self-evident usability aspects but also on issues that they had come across when authentically using the app in daily life. This approach also enabled them to reflect on the effect using the patient portal had on their care management, which would have not been possible in a laboratory setting. However, a follow-up study to evaluate phellow in another medical context is planned and will include a measurement of the app’s usability in a laboratory setting (e.g., eye-tracking [50,51]).

Still, some limitations must be acknowledged. Although mixed-methods design have been recommended for evaluations of mHealth applications [52], our mixed-methods study design produced contradictory data with a high quantitative usability rating on the one hand, but also usability issues derived from the interviews on the other hand. We believe this can be attributed to both the nature of the SUS, which determines a point estimate based on only 10 items, and to the nature of our qualitative interviews. In these interviews, we aimed to discover all the usability issues that might occur and therefore asked in-depth questions, which led us to discover a wide range of usability aspects.

Our sample might include patients with greater interest in mobile applications in general. Being aware of this risk, the perspectives of non-users were integrated. To test phellow in a real-life setting, new users as well as long-term users participated in the study. This might have led to a recall bias, especially when addressing onboarding and registration aspects.

Only one hospital was involved in this study, limiting generalizability. However, since the use of an app to access medical records is a rather new concept in Germany, these data will serve as important information for medical institutions intending to develop similar features.

## 5. Conclusions

The results presented in this paper showed that usability issues such as complicated registration processes can impede patient portal adoption, even though a standardized usability questionnaire showed a good usability rating. In a follow up study, we will assess the app’s usability in a laboratory setting to gain further understanding on this topic.

Our study represents a sound methodologically but nevertheless easily reproducible way to gain insights into the usability and feasibility of a mobile patient portal and provides valuable recommendations for the development of mobile patient portals. We encourage researchers, developers, and HCPs to assess their EHR tools/patient portals for usability or feasibility issues in order to improve their system and to better support patients in actively managing their condition.

## Figures and Tables

**Table 1:** Participant characteristics of the quantitative measures (*n* = 31).

Characteristic	*n* (%) or Mean (SD), Range
Age in years, mean (SD), range	53 (11.9), 27–71
**Gender, *n* (%)**	
Female	9 (29)
**Education, *n* (%)**	
University degree	4 (12.9)
High school diploma	9 (29)
Intermediate secondary education	15 (48.4)
Lower secondary education	3 (9.7)
**Frequency of phellow use, *n* (%)**	
Daily or several times per week	3 (9.7)
Once per week	10 (32.3)
Once per month	18 (58.1)
**Duration of phellow use, *n* (%)**	
Less than one month	1 (3.2)
Approx. one month	3 (9.7)
2–6 months	4 (12.9)
6–12 months	6 (19.4)
Longer than 12 months	17 (54.8)

**Table 2:** Participant characteristics of the qualitative measures (*n* = 23).

Characteristic	*n* (%) or mean (SD), Range
**Patients: Non-users of app (*n* = 5)**	
Age in years, mean (SD), range	50.2 (19.4), 26–67
Gender: male	5 (100)
Treatment duration at HTx outpatient clinic in years, mean (SD), range	12.8 (9.0), 0–26
**Patients: Users of app (*n* = 11)**	
Age in years, mean (SD), range	50.6 (11.2), 33–69
Gender: female	4 (36.4)
Treatment duration at HTx outpatient clinic in years, mean (SD), range	8.9 (5.7), 0–20
Duration of *phellow* use in years, mean (SD), range	1 (0.8), 0–2
**HCPs (*n* = 7)**	
Age in years, mean (SD), range	33.9 (11.8), 21–60
Gender: female	5 (71.4)
Profession: Physician, *n* (%)	3 (42.9)
Years of practice in HTx outpatient clinic, mean (SD), range	4.4 (5.3), 0–16

**Table 3:** Recommendations on app usability and helpful functionalities.

Recommendations on App Usability	Recommendations on Functionalities
**User interface**	**Interaction and information**
Chronically plausible presentation	Access to full record
Automated update or push notification	Communication module
App customization (font size, font style)	Information
Filtering	Patient forum
Additional access via web browser	Progress display
**Ease of use**	Record transfer
Simplified registration and log-in	Read confirmation
	**Organization of therapy**
	Appointments
	Medication refill requests
	Medication plan
	Reminder function
	Shared access for family/care takers
	**Patient-reported data**
	Transfer of vital signs
	Document upload

## Data Availability

The data presented in this study are available on reasonable request from the corresponding author. The data are not publicly available due to privacy protection reasons.

## Supplementary Materials

The following supporting information can be downloaded at: https://www.mdpi.com/article/10.3390/life12081204/s1, Additional file S1: COREQ Checklist; Additional file S2: Interview guide; Additional file S3: Sociodemographic Questionnaire; Additional file S4: Definition of themes.

## References

[1] Blumenthal D., Squires D. (2015). Giving patients control of their EHR data. J. Gen. Intern. Med..

[2] Blumenthal D., Tavenner M. (2010). The “meaningful use” regulation for electronic health records. N. Engl. J. Med..

[3] El-Kareh R., Gandhi T.K., Poon E.G., Newmark L.P., Ungar J., Lipsitz S., Sequist T.D. (2009). Trends in primary care clinician perceptions of a new electronic health record. J. Gen. Intern. Med..

[4] Delbanco T., Walker J., Bell S.K., Darer J.D., Elmore J.G., Farag N., Feldman H.J., Mejilla R., Ngo L., Ralston J.D. (2012). Inviting patients to read their doctors’ notes: A quasi-experimental study and a look ahead. Ann. Intern. Med..

[5] Esch T., Mejilla R., Anselmo M., Podtschaske B., Delbanco T., Walker J. (2016). Engaging patients through open notes: An evaluation using mixed methods. BMJ Open.

[6] Ross S.E., Lin C. (2003). The effects of promoting patient access to medical records: A review. J. Am. Med. Inform. Assoc..

[7] Woods S.S., Schwartz E., Tuepker A., Press N.A., Nazi K.M., Turvey C., Nichol W.P. (2013). Patient experiences with full electronic access to health records and clinical notes through the My HealtheVet Personal Health Record Pilot: Qualitative study. J. Med. Internet Res..

[8] Wright E., Darer J., Tang X., Thompson J., Tusing L., Fossa A., Delbanco T., Ngo L., Walker J. (2015). Sharing physician notes through an electronic portal is associated with improved medication adherence: Quasi-experimental study. J. Med. Internet Res..

[9] Bell S.K., Mejilla R., Anselmo M., Darer J.D., Elmore J.G., Leveille S., Ngo L., Ralston J.D., Delbanco T., Walker J. (2017). When doctors share visit notes with patients: A study of patient and doctor perceptions of documentation errors, safety opportunities and the patient–doctor relationship. BMJ Qual. Saf..

[10] Walker J., Leveille S., Bell S., Chimowitz H., Dong Z., Elmore J.G., Fernandez L., Fossa A., Gerard M., Fitzgerald P. (2019). OpenNotes after 7 years: Patient experiences with ongoing access to their clinicians’ outpatient visit notes. J. Med. Internet Res..

[11] Zanaboni P., Kummervold P.E., Sørensen T., Johansen M.A. (2020). Patient use and experience with online access to electronic health records in Norway: Results from an online survey. J. Med. Internet Res..

[12] Graham T.A.D., Ali S., Avdagovska M., Ballermann M. (2020). Effects of a web-based patient portal on patient satisfaction and missed appointment rates: Survey study. J. Med. Internet Res..

[13] Mack C., Terhorst Y., Stephan M., Baumeister H., Stach M., Messner E.-M., Bengel J., Sander L.B. (2021). “Help in a heartbeat?”: A systematic evaluation of mobile health applications (apps) for coronary heart disease. Int. J. Environ. Res. Public Health.

[14] Jamari J., Ammarullah M.I., Santoso G., Sugiharto S., Supriyono T., Prakoso A.T., Basri H., van der Heide E. (2022). Computational Contact Pressure Prediction of CoCrMo, SS 316L and Ti6Al4V Femoral Head against UHMWPE Acetabular Cup under Gait Cycle. J. Funct. Biomater..

[15] Schrem H., Barg-Hock H., Strassburg C.P., Schwarz A., Klempnauer J. (2009). Aftercare for Patients With Transplanted Organs. Dtsch. Ärzteblatt.

[16] Vega E., Schroder J., Nicoara A. (2017). Postoperative management of heart transplantation patients. Best Pract. Res. Clin. Anaesthesiol..

[17] Söderlund C., Rådegran G. (2015). Immunosuppressive therapies after heart transplantation—The balance between under- and over-immunosuppression. Transpl. Rev..

[18] Müller J., Ullrich C., Poss-Doering R. (2020). Beyond known barriers—Assessing physician perspectives and attitudes toward introducing open health records in germany: Qualitative study. J. Particip. Med..

[19] Schrahe D. (2021). Die ePA vor dem Hintergrund der Gesetzgebung—Der eigenwillige deutsche Weg TT—EHR against the background of the legislation—The unconventional german way. Gesundh. Qual..

[20] Bergh B., Heinze O., Rebscher H., Kaufmann S. (2017). Elektronische Gesundheits- und Patientenakten als digitale Basiskomponenten vernetzter Gesundheit. Digitalisierungsmanagement in Gesundheitssystemen.

[21] Ose D., Kunz A., Pohlmann S., Hofmann H., Qreini M., Krisam J., Uhlmann L., Jacke C., Winkler E.C., Salize H.-J. (2017). A Personal Electronic Health Record: Study Protocol of a Feasibility Study on Implementation in a Real-World Health Care Setting. JMIR Res. Protoc..

[22] Heinze O., Schneider G. (2019). Persönliche Elektronische Patientenakte: Die Digitalisierung der Patient Journey. Kma-Klin. Manag. Aktuell.

[23] Universitätsklinikum Heidelberg (2020). Nachsorge: Neue App für Patienten nach Herztransplantation [Internet]. https://www.kma-online.de/aktuelles/it-digital-health/detail/neue-app-fuer-patienten-nach-herztransplantation-a-42649.

[24] Tong A., Sainsbury P., Craig J. (2007). Consolidated criteria for reporting qualitative research (COREQ): A 32-item checklist for interviews and focus groups. Int. J. Qual. Health Care.

[25] Brooke J., Jordan P.W., Thomas B., Weerdmeester B.A., McClelland I.L. (1996). SUS—A quick and dirty usability scale. Usability Evaluation in Industry.

[26] Bangor A., Kortum P.T., Miller J.T. (2008). An empirical evaluation of the system usability scale. Int. J. Hum. Comput. Interact..

[27] Brooke J. (2013). SUS: A retrospective. J. Usability Stud..

[28] Bangor A., Staff T., Kortum P., Miller J., Staff T. (2009). Determining what individual SUS scores mean: Adding an adjective rating scale. J. Usability Stud..

[29] Lewis J.R., Sauro J., Kurosu M. (2009). The factor structure of the system usability scale. Human Centered Design Lecture Notes in Computer Science.

[30] Harris P.A., Taylor R., Thielke R., Payne J., Gonzalez N., Conde J.G. (2009). Research electronic data capture (REDCap)-A metadata-driven methodology and workflow process for providing translational research informatics support. J. Biomed. Inf..

[31] Harris P.A., Taylor R., Minor B.L., Elliott V., Fernandez M., O’Neal L., McLeod L., Delacqua G., Delacqua F., Kirby J. (2019). The REDCap consortium: Building an international community of software platform partners. J. Biomed. Inf..

[32] Braun V., Clarke V. (2006). Using thematic analysis in psychology. Qual. Res. Psychol..

[33] Sauro J., Lewis J.R. (2016). Quantifying the User Experience: Practical Statistics for User Research.

[34] Ammenwerth E., Iller C., Mahler C. (2006). IT-adoption and the interaction of task, technology and individuals: A fit framework and a case study. BMC Med. Inform. Decis. Mak..

[35] Bowen D.J., Kreuter M., Spring B., Cofta-Woerpel L., Linnan L., Weiner D., Bakken S., Kaplan C.P., Squiers L., Fabrizio C. (2009). How We Design Feasibility Studies. Am. J. Prev. Med..

[36] Bevan N., Carter J., Earthy J., Geis T., Harker S. (2016). New ISO standards for usability, usability reports and usability measures. International Conference on Human-Computer Interaction.

[37] Ceaparu I., Lazar J., Bessiere K., Robinson J., Shneiderman B. (2004). Determining causes and severity of end-user frustration. Int. J. Hum. Comput. Interact..

[38] Jibb L.A., Stevens B.J., Nathan P.C., Seto E., Cafazzo J.A., Johnston D.L., Hum V., Stinson J.N. (2017). Implementation and preliminary effectiveness of a real-time pain management smartphone app for adolescents with cancer: A multicenter pilot clinical study. Pediatric Blood Cancer.

[39] Avdagovska M., Ballermann M., Olson K., Graham T., Menon D., Stafinski T. (2020). Patient portal implementation and uptake: Qualitative comparative case study. J. Med. Internet Res..

[40] Baudendistel I., Winkler E., Kamradt M., Brophy S., Längst G., Eckrich F., Heinze O., Bergh B., Szecsenyi J., Ose D. (2017). Cross-sectoral cancer care: Views from patients and health care professionals regarding a personal electronic health record. Eur. J. Cancer Care.

[41] Walker J., Leveille S., Ngo L., Vodicka E., Darer J., Dhanireddy S., Elmore J.G., Feldman H.J., Lichtenfeld M.J., Oster N. (2011). Inviting patients to read their doctors’ notes: Patients and doctors look ahead: Patient and physician surveys. Ann. Intern. Med..

[42] Ross S.E., Todd J., Moore L.A., Beaty B.L., Wittevrongel L., Lin C.T. (2005). Expectations of patients and physicians regarding patient-accessible medical records. J. Med. Internet Res..

[43] Kayyali R., Peletidi A., Ismail M., Hashim Z., Bandeira P., Bonnah J. (2017). Awareness and Use of mHealth Apps: A Study from England. Pharmacy.

[44] Tsiknakis M., Kouroubali A. (2009). Organizational factors affecting successful adoption of innovative eHealth services: A case study employing the FITT framework. Int. J. Med. Inform..

[45] Ross J., Stevenson F., Lau R., Murray E. (2016). Factors that influence the implementation of e-health: A systematic review of systematic reviews (an update). Implement. Sci..

[46] Ose D., Baudendistel I., Pohlmann S., Winkler E.C., Kunz A., Szecsenyi J. (2017). Persönliche Patientenakten im Internet. Ein narrativer Review zu Einstellungen, Erwartungen, Nutzung und Effekten. Z. Für Evidenz Fortbild. Und Qual. Im Gesundh..

[47] Baudendistel I., Winkler E., Kamradt M., Brophy S., Längst G., Eckrich F., Heinze O., Bergh B., Szecsenyi J., Ose D. (2015). The patients’ active role in managing a personal electronic health record: A qualitative analysis. Supportive Care Cancer.

[48] Tieu L., Sarkar U., Schillinger D., Ralston J.D., Ratanawongsa N., Pasick R., Lyles C.R. (2015). Barriers and facilitators to online portal use among patients and caregivers in a safety net health care system: A qualitative study. J. Med. Internet Res..

[49] Walker J., Delbanco T. (2013). Interval examination: Moving toward open notes. J. Gen. Intern. Med..

[50] Zhou L., Dealmeida D., Parmanto B. (2019). Applying a user-centered approach to building a mobile personal health record app: Development and usability study. JMIR mHealth uHealth.

[51] Cho H., Powell D., Pichon A., Kuhns L.M., Garofalo R., Schnall R. (2019). Eye-tracking retrospective think-aloud as a novel approach for a usability evaluation. Int. J. Med. Inform..

[52] Holl F., Kircher J., Swoboda W.J., Schobel J. (2021). Methods used to evaluate mhealth applications for cardiovascular disease: A quasi-systematic scoping review. Int. J. Environ. Res. Public Health.

