# Supplementary material for: Mobile Access to Medical Records in Heart Transplantation Aftercare: Mixed-Methods Study Assessing Usability, Feasibility and Effects of a Mobile Application
Source: Life (Basel). 2022 Aug 8;12(8):1204. doi: 10.3390/life12081204 (PMC9410472; doi:10.3390/life12081204)
Supplement: Supplementary file 1 [file life-12-01204-s001.zip › Additional file S2_interview guide.pdf]

Additional file S2: Translated interview guides

Interview guide for active users (patients)

Thank you for taking time for this interview!

What you would think if you had access to all your medical records here in the hospital? *Optional*: What health apps do you use, besides *phellow*? (i.e. from your health insurance provider, apps for activity tracking…)

Could you please describe how you first heard about the *phellow* app?

How exactly was the app offered to you?

Optional: What problems did you encounter and what went well?

Optional (if installation was performed by the IT team): If you had to install the app yourself – what would that have been like for you?

Thinking back to the start: How well did you understand what the app can be used for?

How easy or how hard did you find using the app?

Optional: What was especially easy, what was especially difficult?

Which functions do you use in the *phellow* app (i.e. viewing drug trough levels, printing/sharing/sending drug trough levels)?

Optional:

- To what extent have you already reviewed doctor's notes?
- How easy or how difficult was it for you to access your drug trough levels?

What do you think about the app's design?

Optional: How do you like the color arrangement? The font? The font's readability?

What do you think about the navigation in the app?

Optional: How do you like the menu? How easy is it for you to navigate in the app?

What has changed for you since you have been using the app?

Optional: i.e. in coping with your disease or in everyday life

How would you describe the effort when using the app?

To what extent would you recommend the app to other patients? Why?

Let’s presume we were to introduce the app to all patients here. What would you think about that?

Let’s presume you could build the perfect *phellow* app. What would you wish for?
Optional: You’re free to be creative – what should the *phellow* app ideally be able to do?
Optional: You’ve already mentioned a few new functions. Which function would be most important, which would be least important?

What further recommendations on the app do you have?
What further thoughts on this topic would you like to share?

Thank you so much for the conversation and your interesting opinions!

Interview guide for non-users (patients)

Thank you for taking time for this interview!

What you would think if you had access to all your medical records here in the hospital? *Optional*: What health apps do you use, besides *phellow*? (i.e. from your health insurance provider , apps for activity tracking…)

Could you please describe how you first heard about the *phellow* app?

How exactly was the app offered to you?

Optional: What problems did you encounter and what went well?

Optional (if installation was performed by the IT team): If you had to install the app yourself – what would that have been like for you?

Would you please describe how testing the app went for you?

Thinking back to the start: How well did you understand what the app can be used for?

How easy or how hard did you find using the app?

Optional: What was especially easy, what was especially difficult?

Which functions do you test in the *phellow* app (i.e. viewing drug trough levels, printing/sharing/sending drug trough levels)?

Optional:

- To what extent have you already reviewed doctor's notes?
- How easy or how difficult was it for you to access your drug trough levels?

What did you think about the app’s design?

Optional: How do you like the color arrangement? The font? The font’s readability?

What did you think about the navigation in the app?

Optional: How do you like the menu? How easy is it for you to navigate in the app?

How would you describe the effort when testing the app?

Why did you decide not to use the app?

Optional: What were the main reasons for this decision?

To what extent would you recommend the app to other patients? Why?

Let’s presume we were to introduce the app to all patients here. What would you think about that?

Let’s presume you could build the perfect *phellow* app. What would you wish for?
Optional: You’re free to be creative – what should the *phellow* app ideally be able to do?
Optional: You’ve already mentioned a few new functions. Which function would be most important, which would be least important?

Let’s presume you could access your drug trough levels differently, i.e. via your PC. What do you think about that?

What further recommendations on the app do you have?
What further thoughts on this topic would you like to share?

Thank you so much for the conversation and your interesting opinions!

Interview guide for HCPs

Thank you for taking time for this interview!

In general, what do you think about giving patients access to their medical records?

Optional: What advantages and disadvantages do you see with this approach?

Could you please describe how the implementation of *phellow* went?

Optional: In the beginning, how and when did you receive information about *phellow*?

Optional: What (other) problems did you notice in the implementation phase?

How would you describe the patients' interest in the app?

Which patients do you offer the app?

What experiences with *phellow* were reported from patients to you and your colleagues?

What are the experiences you and your colleagues have made with *phellow*?

Optional:

- What experiences did physicians report?
- And non-physician team members?

How does the app fit in with the clinic workflow?

Let's presume we were to introduce the app to all patients here. What would you think about that?

Optional:

- What has to be changed so everyone could use the app?
- What chances and risks do you anticipate?

Which additional functions would you wish for in *phellow*?

Optional: You're free to be creative – what should the *phellow* app ideally be able to do?

Optional: You've already mentioned a few new functions. Which function would be most important, which would be least important?

What further recommendations on the app do you have?

What further thoughts on this topic would you like to share?

Thank you so much for the conversation and your interesting opinions!
