# Supplementary material for: Mobile Access to Medical Records in Heart Transplantation Aftercare: Mixed-Methods Study Assessing Usability, Feasibility and Effects of a Mobile Application
Source: Life (Basel). 2022 Aug 8;12(8):1204. doi: 10.3390/life12081204 (PMC9410472; doi:10.3390/life12081204)
Supplement: Supplementary file 1 [file life-12-01204-s001.zip › Additional file S3_sociodemographic questionnaires.pdf]

Additional file S3: Translated sociodemographic questionnaires for interviews

Questionnaire for non-users

Interview No.: ______________

Personal Details:

Gender:

- ☐ Female
- ☐ Male
- ☐ Diverse

Age: ______________

Since when have you been receiving treatment at the heart transplantation outpatient clinic in Heidelberg?

____

Interview No.: ______________

Personal Details:

Gender:

- ☐ Female
- ☐ Male
- ☐ Diverse

Age: ______________

Since when have you been receiving treatment at the heart transplantation outpatient clinic in Heidelberg?

____

Details on app use:

Since when have you been using the *phellow* app? ____________________________

How often do you use the *phellow* app?

- ☐ Daily
- ☐ Weekly
- ☐ Monthly
- ☐ Half-yearly
- ☐ Less frequently than half-yearly

Interview No.: ______________

Personal Details:

Gender:

- ☐ Female
- ☐ Male
- ☐ Diverse

Age: ______________

Occupational details:

Occupation: ____________________________

Since when have you been working at the heart transplantation outpatient clinic in Heidelberg?

____
