# Supplementary material for: Mobile Access to Medical Records in Heart Transplantation Aftercare: Mixed-Methods Study Assessing Usability, Feasibility and Effects of a Mobile Application
Source: Life (Basel). 2022 Aug 8;12(8):1204. doi: 10.3390/life12081204 (PMC9410472; doi:10.3390/life12081204)
Supplement: Supplementary file 1 [file life-12-01204-s001.zip › Additional file S4_Definition of themes.pdf]

Additional file S4: Definition of themes

Theme: Feasibility

Subthemes	*Definition*	*Text samples from transcripts*
Task	Codes referring to tasks and interactions with the app *phellow* and the accompanying infrastructure.	*[…] at the moment, I only know about the drug level changes. If there is something and something has changed, then there is the follow-up status, I click on it, I am asked again if I am sure to save changes and then it automatically goes to the app.* [medical staff #7, TP 24]
		And for us, as employees, it makes it easier, especially for the drug levels. Everything goes much faster. And you don't have to call anymore. That already takes a lot of work off. Just calling. Especially when patients don't answer the phone and there is a change [in drug levels]. And that's when you start to worry a little bit. Especially if it's Friday, because you can of course not call on Saturday or Sunday. And then the patient doesn't have their newest dose and that is of course sometimes bad. Or if you leave a voicemail which you shouldn't do either. That's why I Think the app is really good. [medical staff #12, TP 4]
		I2: And you think these documents are critical, because they have to be explained to the patient in context […]?<br><br>*P: Exactly. […] But if you just give them the [organ transplantation] conference decisions, that's…[…] and they're saying "he won't get a new organ, he's too old, doesn't fit anymore", that's nothing that they should read just like that, at the coffee table.* [medical staff #8, TP 81]
		Which means that easy instructions or a manual to give to the patients would be very useful. There's also no hotline where people can ask if they have problems, for instance. Especially because we have older patients […]. – This is where I see a big problem. Because the app itself is well liked. But it has some small issues which should be solved. Then, it would probably be a lot better. [physician #9, TP 20]

Individuals	Codes referring to participants' experiences, requirements, or views regarding further implementation of *phellow* into (routine) care.	*I would recommend it, but I don't think it works for everyone. So I think a lot of people, including elderly or people who can't cope with smartphones at all, I think that would be difficult. In general, I would recommend it to everybody. For those, who are interested, it is not a problem. […] And it is the future. And that's why one should deal with it. And I think that – if it goes well and is right – it is a relief for the clinic as well. It depends on that. I think it would be good and I would also recommend it to others.* [user #17, TP 74]
		What kind of errors can occur, what are the most common errors… because patients can't even describe it. […] And that's why it would certainly be good if I was more involved. Perhaps I can then give patients more information when problems arise. And […] if one could train us as well and tell us what error sources are possible or likely, where we can support [patients] and where we can only refer them to an address… And then, we also know more. [medical staff #06, TP 70]
		But other than that, I don't really look at the physician's letters, because I can't comprehend them anyway. [user #5, TP 4]
		And they [HCP] also have a note in my record which says "app doesn't work, please call [to report drug level changes]". And afterwards nobody cared for that. I asked them about it once, but then they said they didn't have time for this now. Another time they told me to contact IT. I also googled. But from all these pages, I didn't know who would be the right person to contact. [non-user #1, TP 24]
Technology	Codes referring to participants' thoughts or perceptions about the technical aspects surrounding the system/*phellow*.	*Maybe the system will be overstrained in processing that much data?* [non user #2, TP 56]

Theme: Usability

Subthemes	*Definition*	*Text samples from transcripts*
Satisfaction	The level of comfort people experience in achieving their goals	*I think it's good that it [the app] exists. Of course, you can always continue development. It's an important topic, too. But I think it's a good thing.* [user #3, TP 38]
		And if they [the patients] have the app then I assume that it works. It works for many people, no question, but I never have control, as I said. [medical staff #6, TP 50]
		Well, it can always happen that an iPhone or a phone crashes. That you can't access the app. An alternative is still important, calling them or something. [non-user #23, TP 50]
		I like that you can get blood test results faster. It sometimes took quite a long time to get new dose recommendations for the dosage of drugs. And that you can shorten the time with it [the app]. And then I thought "Of course I'll do it, I'll try it out". [user #22, TP 12]
Efficiency	The extent to which people expend resources in achieving their goals	*I: Okay. Let's go back to the beginning for a moment: How well did you understand what to do with the app?*<br><br>*P: Actually, good from the beginning. That was never a problem.* [user #20, TP 21-22]
		It's also simple built, it's not that you don't know where to press a button or something. So, in general, easy. [non-user #2, TP 20]
		The registration was not so easy. I often got a message that the password was wrong, and then "could not be connected", and then I did everything and at some point I was so annoyed that I just stopped, and the next day it suddenly worked. [user #16, TP 16]
		It's a time saver, for patients and also for the people from the other side, nurses, physicians… [user #4, TP 42]
		Because I think the advantages are that the patients don't have to call us. They get the message directly, we don't lose any time. I think that makes a lot of sense. Especially for immunosuppression levels. [physician #9, TP 26]

Effectiveness	Whether people can complete their tasks and achieve their goals	*Yes, at some point, there was a letter in the app, but then nothing for a long time. So I have one physician's letter in the app. I think its from July or August. And all the other letters are not in there.* [user #17, TP 18]
		[…] what I find totally wrong is that the date from when the initial document was created is the first that you see, and if there are changes it's not shown anywhere. There is no push notification, there is no change in the UI, it just says the original date of creation and that's stupid in my opinion because I don't care when something is uploaded for the first time, I just want to see when there is something new and that I'm informed that there are news. [user #4, TP 14]
		A big problem is that the app doesn't work for many patients. Why it doesn't work is not entirely clear to me. […] Patients want to install the app, cannot not use it, put it aside and are disappointed. [physician #9, TP 20]
		From what I've seen so far, some patients are very interested in seeing things, in not being dependent on someone answering the phone. They rather have a little more control and access it themselves. [physician #15, TP 18]
		Or, often when I prepare my medication, [I think] "was it like this or like that?", because things have changed. And, of course, I can then just quickly check my app and see what I've got. That's great. [user #14, TP 38]
		I checked it out a little bit, the menu, and it was actually easy for me to understand. [user #3, TP 10]
		I actually find it quite appealing. Pleasant colors. Design is clear, you are not misguided by too much information or something. It's reduced to the essential. [user #16, TP 26]

Theme: Recommendations on app changes

Subthemes	*Definition*	*Text samples from transcripts*
Recommendations on app usability	Recommendations on improving usability of the app	*Of course, it would be better if you could open it [the app] and immediately see "Ah, the first one up there is the newest", and then just click on it.* [user #19, TP 62]
		Maybe that the data from a year ago are visible here in the app, and all other data you can just hide. [user #4, TP 54]
Recommendations on app functionalities	Recommendations on enhancing the functions of the app	*It would be important if the blood values were in, for example. […] and the physician's letters and the examination results, like echo[cardiogram], that's not in there at all.* [user #14, TP 56]
		Or if you have any questions, for example, and you can't reach anyone by telephone – especially during corona, that was sometimes a major problem – then I don't have the option to leave a message via the app, for example, which are then displayed and they can respond directly. [user #10, TP 2]
		[…] you often come across technical terms and then you have to search the Internet. But you never know how trustworthy the information is. […] And that's why the explanation of technical terms would actually be quite good, yes. [user #22, TP 84]
		What might be useful, if you could connect users among themselves. That they could help each other. That would probably a good idea. [physician #9, TP 60]
		It would be important if the blood values were in the app, for example. That I can also compare, what has become worse, how was it last time… that I can compare. [user #14, TP 56]
		And also, for example, I send my blood values from the GP […], that I could also transfer them. Then everything would be together. My blood values would also be in here from the physician there. [user #17, TP 58]
		What you could perhaps also do is provide feedback to us as to whether the person, who has the app, has seen it. […] That you see a check mark, "read", "arrived". [physician #9, TP 60]

Theme: Effects on self-monitoring

Subthemes	*Definition*	*Text samples from transcripts*
independence	Descriptions of independence compared to earlier processes	*Then you always have your data with you, you don't have to carry loose papers anymore, so you can always show that to other physicians. [user #4, TP 42]*
reassurance	Ability to check correct medication and dosage	*Sometimes, I forget it but in the evening, before I take my pill, I used to think "Oh, maybe I should have called". Now, I can just look it up. Or, when I prepare my medication, [I think] "was it like this or like that?. And, of course, I can then just quickly check my app and see. That's great." [user#14, TP38]*
Loss of personal contact	Descriptions of less personal contact and its implications	*…I can't write something within the app. If it was an email, I would be able to ask a question and receive an answer. But here (in the app), I only get the results and can't get in touch through the app. Like with emails, where I can write something and ask questions. [user#17, TP52]*
